# Equine Arteritis Virus in Monocytic Cells Suppresses Differentiation and Function of Dendritic Cells

**DOI:** 10.3390/v15010255

**Published:** 2023-01-16

**Authors:** Nathifa A. Moyo, Dave Westcott, Rachel Simmonds, Falko Steinbach

**Affiliations:** 1 Animal and Plant Health Agency, Virology Department, Addlestone KT15 3NB, UK; 2 Faculty of Health and Medical Sciences, University of Surrey, Guildford GU2 7XH, UK

**Keywords:** equine arteritis virus, monocyte-derived dendritic cells, virology, immunology

## Abstract

Equine viral arteritis is an infectious disease of equids caused by equine arteritis virus (EAV), an RNA virus of the family Arteriviridae. Dendritic cells (DC) are important modulators of the immune response with the ability to present antigen to naïve T cells and can be generated in vitro from monocytes (MoDC). DC are important targets for many viruses and this interaction is crucial for the establishment—or rather not—of an anti-viral immunity. Little is known of the effect EAV has on host immune cells, particularly DC. To study the interaction of eqDC with EAV in vitro, an optimized eqMoDC system was used, which was established in a previous study. MoDC were infected with strains of different genotypes and pathogenicity. Virus replication was determined through titration and qPCR. The effect of the virus on morphology, phenotype and function of cells was assessed using light microscopy, flow cytometry and in vitro assays. This study confirms that EAV replicates in monocytes and MoDC. The replication was most efficient in mature MoDC, but variable between strains. Only the virulent strain caused a significant down-regulation of certain proteins such as CD14 and CD163 on monocytes and of CD83 on mature MoDC. Functional studies conducted after infection showed that EAV inhibited the endocytic and phagocytic capacity of Mo and mature MoDC with minimal effect on immature MoDC. Infected MoDC showed a reduced ability to stimulate T cells. Ultimately, EAV replication resulted in an apoptosis-mediated cell death. Thus, EAV evades the host anti-viral immunity both by inhibition of antigen presentation early after infection and through killing infected DC during replication.

## 1. Introduction

Equine arteritis virus (EAV), now classified as *Alphaarterivirus equid*, is the causative agent of Equine viral arteritis (EVA) and was a prototype of the Arterivirus family. It is now the sole member of the genus Alphaarterivirus in the subfamily *Equarterivirinae* [1]. Like other members of the Arterivirus family, EVA is subject to rapid evolution and several genetic clades can be distinguished, albeit only one serogroup exists [2,3]. Re-emergence of EVA is of concern for the equine industry, and events reported in the USA in 2006/07 [4], France, 2007 [5], Argentina, 2010 [6], and at a smaller scale in other countries including the UK [3] demonstrate that EVA remains a global problem [7]. To understand the occasional outbreaks of severe clinical disease through highly pathogenic variants, it is important to understand how EAV interacts with its host cells to cause disease. 

EVA is a systemic infection where the clinical picture ranges from asymptomatic infection to severe disease. The outcome of the infection is dependent on the virus strain and immune status of the horse. It is long known that EAV replicates in monocytes (Mo) and macrophages (MΦ) and endothelial cells in vivo [8,9] but the related dendritic cells (DC), which are key for initiating an anti-viral immune response and represent an ideal target for viruses seeking to evade or delay the immune response, have not been studied for the interaction with DC.

Dendritic cells (DC) are crucial to host immunity. As antigen presenting cells (APC), they are the only cells that can initiate a primary adaptive immune response [10,11]. The inhibition of DC functions by invading pathogens, such as viruses, can have severe consequences for the establishment of an anti-viral immunity. No studies to date have investigated the effects of EAV on equine DC. Monocyte-derived dendritic cells (MoDC) are a good model that represent inflammatory DC and possess all DC functions [12]. An equine MoDC (eqMoDC) system has been established previously based on phenotype, function, and gene expression [13,14], which can now be used to study equine virus-host interactions. 

To characterize the interaction of EAV with host monocytes and DCs, strains of different genotypes and pathogenicity were used, including the US Bucyrus velogenic strain and an attenuated variant thereof, as well as a UK field strain of lesser pathogenicity. To assess virus infection and replication, qPCR and viral titration assays were employed. The effects of the virus on phenotype and function of cells were assessed using flow cytometry and functional assays. EVA replicates to various degrees in different stages of myeloid cells (monocytes vs. immature and mature DCs) and the replication results in the inhibition of DCs to stimulate immune reactions.

## 2. Materials and Methods

### 2.1. In Vitro Generation of Equine Monocyte-Derived Dendritic Cells

EqMoDC were generated as described in Moyo et al. [14]. Briefly, PBMC were isolated from healthy horses by Ficoll density centrifugation as previously described [13]. Monocytes were further isolated using the monoclonal antibody to human CD14, big 13 clone (Biometec, Greifswald, Germany) also as described [15]. Monocytes were seeded into 24-well flat-bottom tissue culture plates (Greiner bio-one, Stonehouse, UK) at a concentration of 2 × 10^6^ cells per well in 1 mL of RPMI 1640 media (Gibco-Invitrogen, Paisley, UK) supplemented with 10% FCS (Autogen Bioclear, Wiltshire, UK), 0.1 mg/mL of penicillin and streptomycin (Gibco-Invitrogen) and 2% HEPES (Gibco-Invitrogen). Cells were differentiated into immature MoDC (iMoDC) with the addition of 1000 Units/mL and 500 Units/mL of purified recombinant equine GM-CSF and IL-4 (R&D Systems, Abingdon, UK), respectively, and cultured for 5 days [13]. For maturation iMoDC were exposed to a DC maturation cocktail comprising of 20 ng/mL eqTNF-α (R&D Systems) 10 ng/mL eqIL-1β (R&D Systems), 20 µg/mL eqIL-6 (R&D Systems), 1 µg/mL PGE2 (Enzo Life Sciences, Exeter, UK) and 100 ng/mL eqIFN-γ (R&D Systems). 

### 2.2. Virus Strains 

The EAV strains investigated were the velogenic US Bucyrus reference strain [16], an attenuated variant thereof and the UK1 strain. The US Bucyrus velogenic variant was derived as a passage 2 virus of the original Bucyrus isolate and has been shown to cause severe disease in experimentally inoculated horses [17]. The US Bucyrus attenuated strain was prepared from the first passage stock of the velogenic Bucyrus virus by sequential serial passages: 20 times in African green monkey kidney cells (Vero), 3 times in baby hamster kidney cells (BHK-21) and 6 times again in Vero [18,19]. In challenge studies conducted with ponies, the attenuated variant showed no visibly observable illness. The level and duration of nasal virus shedding was very low and infected ponies had neither a detectable viraemia nor rectal virus shedding [19,20]. The UK1 strain was isolated from semen of an infected stallion imported from Poland in 1993. This stallion was responsible for the EAV outbreak in the UK in 1993. It is considered to be of low pathogenicity based on the acute clinical signs observed from horses [21]. A UV inactivated virus preparation was included in all infection assays. For UV inactivation, sterile petri dishes (φ 60 mm) (Thermo Scientific, Surrey, UK) were coated with FCS for 30 min to prevent the virus from adhering to the petri dishes. An amount of 1 mL of virus suspension was exposed to 254 nm of UV wavelengths for 1 h in a class II biological safety cabinet. To ensure the virus had lost its infectivity, virus titration assays were performed and confirmed its inability to infect equine embryonic lung cells (EEL) (kindly provided by the Animal Health Trust, Newmarket, UK). Using qPCR confirmed the presence of virus after UV inactivation. 

Monolayer cultures of EEL were propagated in Minimal Essential Medium (MEM) (Gibco-Invitrogen) supplemented with 10% FCS, 0.1 mg/mL penicillin and streptomycin. Upon infection with EAV, cultures were incubated for 3 days by which time most of the cells had undergone cytopathogenic death. Subsequently, the EAV cultures were frozen at −80 °C overnight and thawed to release virus particles from remaining cells. The virus-cell suspensions were centrifuged at 930× *g* for 10 min at 4 °C to separate the cell debris. The virus supernatants were collected and used to inoculate more EEL cultures, where MEM was subsequently supplemented with 2% FCS for subsequent concentration of the virus. 

Virus suspensions were concentrated using cellulose visking tubes at an exclusion size of 20 kDa (The Scientific Instrument Centre, Hampshire, UK). The virus suspension was added to the tubes, which were submersed in PEG powder at 4 °C until the volume of virus suspension was reduced approximately 50-fold. After concentration, the virus suspension was dialysed, whereby the virus suspension was equilibrated against PBS. Finally, the virus suspensions were filtered with 0.2 μm syringe filters.

### 2.3. Virus Infection of Monocytes and MoDC

Mo and MoDC were inoculated with EAV at MOI of 5 in 24-well tissue culture plates. In parallel, a control group was also exposed to supernatants from EEL cells (mock). Mock supernatants were concentrated and purified in the same manner as the infected supernatants. Mo and MoDC cultures were incubated at 4 °C for 90 min to allow virus attachment to cells. Cells were washed gently three times with cold media and centrifuged at 500× *g* for 2 min after each wash to remove virus inocula. The third media wash was collected for baseline measurements and is referred to as the zero-hour time point. After incubation for various times, cells were harvested for further analysis.

### 2.4. Virus Titration and Detection

EEL cells were trypsinised with 2% Trypsin containing 0.2% Versene (Life Technology-Invitrogen). Cells were counted using a haemocytometer and trypan blue (Life Technologies-Gibco). The cell density was adjusted to 3 × 10^6^ cells in 10 mL of MEM with 2% FCS. The samples to be titrated were serially diluted 10-fold. A total of 100 μL of 3 × 10^4^ EEL cells were dispensed into 96-well microtiter plates, followed by 50 μL of MEM and 25 μL of the respective virus dilution. Each dilution was added to 8 wells (1 column of a microtitre plate), starting with the lowest dilution. To the control wells the addition of 25 μL of MEM with 2% FCS replaced the test sample. Titers were determined after 3 days, and the TCID50/mL determined using the modified Kärber formula [22].

RNA was extracted from cells, using the RNeasy Plus Mini Kit (Qiagen, West Sussex, UK), and first reverse transcribed to cDNA with the SuperScript II First-Strand synthesis system using random hexamer primers (Life Technologies-Invitrogen). RNA extracted from supernatants of infected EEL was used as the positive control in all assays. An ORF1 real-time TaqMan quantitative PCR (qPCR) is established at APHA, UK for the detection of EAV. The primer and probe sequences for this assay are shown in Table 1. 

The master mix was prepared using the QuantiTect Virus Rox Vial Kit (Qiagen). An ORF1 qPCR reaction mix consisted of a 5X QuantiTect PCR buffer, 400 nM each of the forward primer EAVQ2F and the reverse primer EAVQ2R (Metabion International, Martinsried, Germany), 5 pM of the probe EAVQ2P (Metabion International), 50 ng/μL of cDNA and nuclease-free water (Qiagen, West Sussex, UK) to a final reaction volume of 25 μL. The detection of virus was carried out relative to the eukaryotic 18S rRNA to control for sample-to-sample variation [23,24]. The 18S rRNA TaqMan qPCR reaction mix consisted of a 5X QuantiTect PCR buffer, 1X 18s primer-probe mix, 50 ng/μL cDNA and nuclease-free water to a final reaction volume of 25 μL. The cycling conditions for both assays were 95 °C for 10 min and 40 cycles of 95 °C for 30 s followed by 60 °C for 1 min. The detection limit of the ORF1 assay was 10 TCID50/reaction for all strains used here. The amplification efficiencies of the EAV ORF1 and 18S genes were close to 100% and within 5% of each other, which fulfilled the conditions of the Livak method. Hence, the relative detection of the EAV ORF1 gene was analyzed using the 2-∆∆Ct [25]. 

### 2.5. Phenotypic Analysis of Infected Cells by Flow Cytometry and qPCR

The analysis of cell viability was included in phenotyping and functional flow cytometric assays. Using the live/dead fixable near-IR (infra-red) or violet dead cell stain kits (Fisher Scientific, Loughborough, UK), gates were drawn around the live cell population acquired on the logarithmic scale of the fluorescent dye signal versus the linear scale of the forward side scatter area (FSC-A). The live cell population was then assessed on the linear scales of side scatter area (SSC-A) versus FSC-A to gate the target population. Duplex cells were then excluded from the target population by gating the single cells displayed on the linear scales of SSC-A versus side scatter height (SSC-H). This population of interest was used for further analysis of all fluorescent antibodies on a logarithmic scale. 

To investigate the expression of surface markers cells were analysed using anti-human CD14 mAb big 13, anti-human CD206 clone 3.29B1.10 (Beckman Coulter, High Wycombe, UK), anti-human CD83 clone HB15a (Beckman Coulter), anti-human CD86 clone IT2.2 (Becton Dickinson, Oxford, UK) and an anti-horse MHC II clone EqT2 (VMRD, Pullman, WA, USA), all as described before [14]. Stained cells were analyzed using a MACSQuant Analyzer and MACSQuant software (Miltenyi Biotec, Bergisch Gladbach, Germany). 

Separately, apoptotic assays were performed using the Annexin V Apoptosis Detection kit (Becton Dickinson) following the manufacturer’s instructions. Briefly, cells were washed twice with cold PBS and resuspended in 1× binding buffer at a concentration of 1 × 10^6^ cells/mL. A quantity of 100 μL (1 × 10^5^ cells) of cell suspension was added to a FACS tube followed by the addition of 5 μL of both Annexin V-FITC and 7-AAD. Some 7-Amino-Actinomycin (7-AAD) (Becton Dickinson, Wokingham, UK) was used to allow the identification of both early- and late-stage apoptotic cells. The cells were gently mixed and incubated for 15 min at room temperature in the dark. After incubation, 400 μL of 1× binding buffer was added to each tube and cells immediately analyzed by flow cytometry.

A selected set of co-stimulatory genes identified in the horse previously, namely PDL1/CD274, PDL2/CD273, B7-H3/CD276 and ICOSL/CD275, was used to assess the effect of infection on their expression levels using qPCR where no mAbs were available [14]. In addition, the expression of selected cytokines (IL-10 and IL-29) was assessed using TaqMan assays developed for this study. Equine-specific primers were designed with Primer3 and the primer sequences are shown in Table 1. The protocol for the qPCR assays and data analysis was carried out as before [14]. Briefly, cDNA synthesis was performed with the SuperScript II First-Strand Synthesis System using random hexamer primers (Invitrogen). qPCR reactions were performed in triplicates. The 18S rRNA TaqMan qPCR was used as the endogenous control (Applied Biosystems, Warrington, UK). The cycling conditions included a denaturation step at 95 °C for 10 min followed by 40 cycles at 95 °C for 15 s and 60 °C for 1 min. PCR was analyzed by relative quantification using the ∆∆Ct method. Statistical analysis here and for other assays was performed using GraphPad Prism 5 software. 

### 2.6. Functional Assays 

The ability of infected MoDC to endocytose APC-labelled OVA (Fisher Scientific, Leicestershire, UK) or phagocytose FITC-labelled FluoSphere carboxylate-conjugated microsphere particles (1.0 μm diameter) (Invitrogen) was assessed by flow cytometry following previously published protocols [26,27,28]. Briefly, infected or control monocytes, iMoDC or mMoDC were washed once and resuspended in RPMI 1640 media at a density of 1 × 10^5^ cells per well of a flat-bottomed 96 well plate (Invitrogen). All plates were incubated on ice for 30 min before adding OVA-APC to a final concentration of 20 μg/mL and FITC-conjugated carboxylate-modified microsphere at a ratio of 5:1 (bead/cell). Cells were incubated at 4 °C (control) and 37 °C for 1 h or 4 h for the endocytic and phagocytic assays, respectively, subsequently washed three times with cold PBS solution (Invitrogen) and re-suspended in PBS for flow cytometric analysis. 

Mixed leukocyte reactions (MLR) were performed as described in Moyo et al. [14]. Briefly, equine T lymphocytes were enriched using anti-horse CD5, clone CVS5 (Serotec, Kidlington, UK) and magnetically sorted. Infected or control MoDC from one horse were added in graded doses to 5 × 10^5^ CFSE labelled T lymphocytes from another horse. Labelling of cells with CFSE was carried out as previously described [29]. Subsequently, cells were co-cultured at 37 °C for 3 days, before proliferation of T cells was measured by flow cytometry as previously described [10]. 

Primary antigen presentation was measured by incubating graded numbers of infected or control iMoDC at 37 °C for 2 h with 1 mg/mL of LPS-free OVA, which can be considered an antigen horses do not encounter. After incubation, iMoDC were matured overnight with the cocktail as described above. CFSE-labelled T lymphocytes from the same horse were added to the infected mMoDC at a density of 5 × 10^5^ cells and co-cultured at 37 °C. After 4 days, proliferation of live T cells was evaluated by flow cytometry as in the MLR assays. 

## 3. Results

### 3.1. Replication of EAV in Equine Mo and MoDC

Previous studies had established that myeloid cells and in particular macrophages resembled host cells for EAV [2]. It was thus of interest to establish if both monocytes and different stages of DCs were also able to support the replication of EAV and to quantify the replication to allow for comparisons between both host cells and virus strains. The TaqMan qPCR for ORF1 was used to quantify viral RNA as, contrast to other genes that are expressed in nested sets of RNA, it only occurs in the genomic form and the antisense replication intermediate. Thus, its quantification parallels replication. Normalization controlled for differences in cell numbers between cultures of various treatments. While the qPCR assay was used to determine active replication in cells, it was complemented by virus titration to determine the amount of infectious virus released into the cell culture supernatant. 

**Figure 1:**
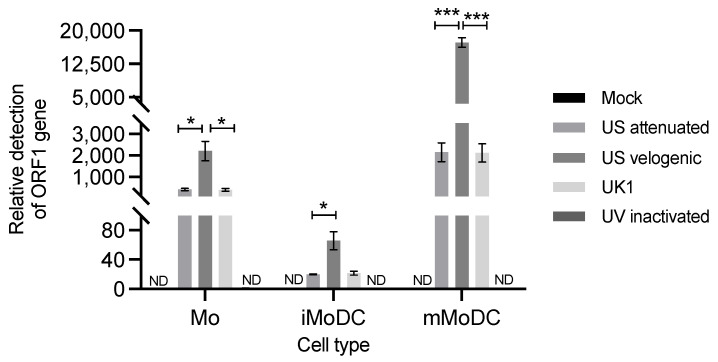
Detection of viral replication in equine Mo and MoDC by qPCR targeting the ORF1 region of EAV. Horse Monocytes, immature MoDC (iMoDC) and mature MoDC (mMoDC) were inoculated at MOI 5 with different preparations of replicative or UV inactivated EAV. RNA was extracted from cells 16 hpi, reverse transcribed into cDNA and EAV replication quantified by qPCR, using 18S rRNA gene as the reference gene. The normalized fold difference ratio was calculated using the 2-ΔΔCt formula with 0 hpi as the comparator. Results were represented as the average fold difference ± SEM (*n* = 9). ND represents no viral detection in mock and UV inactivated infected cells. Statistical significance was determined using a 2-way ANOVA with Bonferroni post-test to compare replicate means. * and *** indicate significant differences between sample means where *p* < 0.005 or 0.0001, respectively.

The ORF1 TaqMan qPCR assay detected viral replication of all strains in all three cell types 16 h post infection. The 0 h post-infection timepoint was used as the calibrator (base line) in the relative quantification, excluding the amount of virus attached to the cells. The relative detection of ORF1 was the highest in mMoDC followed by Mo and the least iMoDC (Figure 1). Hence, mMoDC seemed the most susceptible to EAV whereas iMoDC were the least susceptible. In iMoDC, the overall expression of the ORF1 gene for all strains was low (<100-fold compared to other cells) with no significant differences between strains. The comparison of the relative detection between strains revealed that the velogenic strain had the highest expression of the ORF1 gene. The attenuated and UK1 strains had similar ORF1 expression levels in Mo and MoDC. As expected, the UV inactivated virus did not show any sign of replication. 

**Figure 2:**
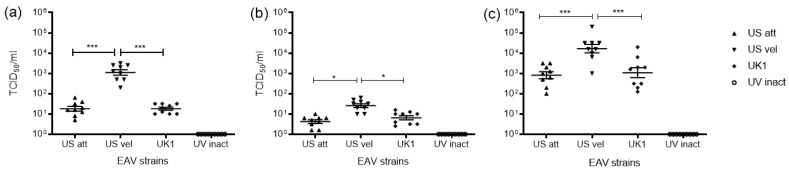
Viral infectivity titers from supernatants of infected Mo and MoDC cultures titrated on EEL. Monocytes (**a**), immature MoDC (iMoDC) (**b**), and mature (mMoDC) (**c**) were inoculated at MOI 5 with different strains of live or UV inactivated EAV. Virus titrations were performed on 0 and 16 hpi supernatants. The TCID50/mL at 0 hpi was subtracted from the TCID50/mL at 16 hpi. For all cells the TCID50/mL was the highest with the Velogenic strain. Overall, the titers were the highest in mMoDC followed by Mo and the least in iMoDC cultures. Results represent the TCID50/mL ± SEM (*n* = 9). A two-tailed paired Student *t* test was used for single comparisons between the 0 and 16 hpi titers (shown in black). A 1-way ANOVA with Dunn’s multiple comparison test was used for comparisons between strains at 16 hpi (shown in red). * and *** indicate significant differences between sample means where *p* < 0.05 and 0.0001, respectively.

Depending on the strain, the virus titers in Mo supernatants ranged from 10^1.0^ to 10^3.0^ TCID50/mL (Figure 2a). Immature MoDC cultures had the lowest virus titers spanning from 10^0.5^ to 10^1.5^ TCID50/mL (Figure 2b), whereas supernatants from mMoDC cultures had the highest titers which ranged from 10^3^.^0^ to 10^4^.^5^ TCID50/mL (Figure 2c). The results of the TCID50 assays are displayed as the difference in infectivity titers between the 0 h and 16 h post infection for each virus strains in Mo and MoDC supernatants. Thus, newly produced virus can be distinguished from the infectious dose that was present after the infection. The differences in titers between EAV strains were significant and in line with the qPCR data, i.e., the Velogenic strain had the highest titers in all cell types, while the attenuated and UK1 strains displayed similar titers. These data confirmed the production of infectious virions as a result of virus replication. For the UV inactivated virus, no titers were obtained, which demonstrated the inability of this preparation to infect cells.

### 3.2. The Effect of EAV on Equine Mo and MoDC Viability

**Figure 3:**
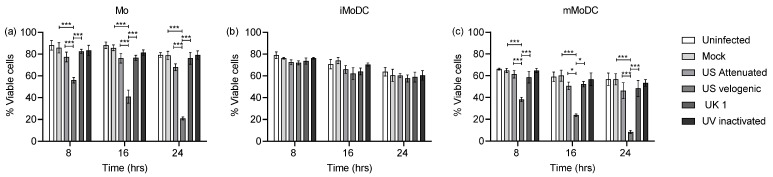
Time kinetics of the viability of EAV infected Mo and MoDC. Mo, immature (iMoDC) and mature (mMoDC) were inoculated at MOI of 5 with different strains of live or UV inactivated EAV. Cultures were incubated 37 °C in 5% CO_2_ for (**a**) 8 h, (**b**) 16 h, and (**c**) 24 h post infection (hpi). Controls included uninfected cultures (media only) and mock infected cultures. Cells were stained with the violet dead cell stain and analysed by flow cytometry. Data are represented as the mean percentage viable cells ± SEM (*n* = 4). Statistical significance was determined using a 2-way ANOVA with Bonferroni posttest to compare replicate means. * and *** indicate significant differences between sample means where *p* < 0.05 and 0.0001, respectively.

Preliminary observation of infected cells over a few days had confirmed that EAV replicated lytically in myeloid cells. It was thus important to determine an early time point during the primary replication cycle to conduct phenotypic and functional studies after infected. Accordingly, cell viability assays were performed at different time points ranging from 8 to 24 h post infection covering the time for a primary EAV replication cycle described in other susceptible cells [30]. The data revealed that already early after infection there were significant differences in the Mo and mMoDC viability upon infection with the Velogenic strain (Figure 3). The percentage of viable Mo and mMoDC here continuously decreased over 24 h post infection to around 10%, (Figure 3c). EAV had no significant effect on the viability of iMoDC until 24 h post infection. 

**Figure 4:**
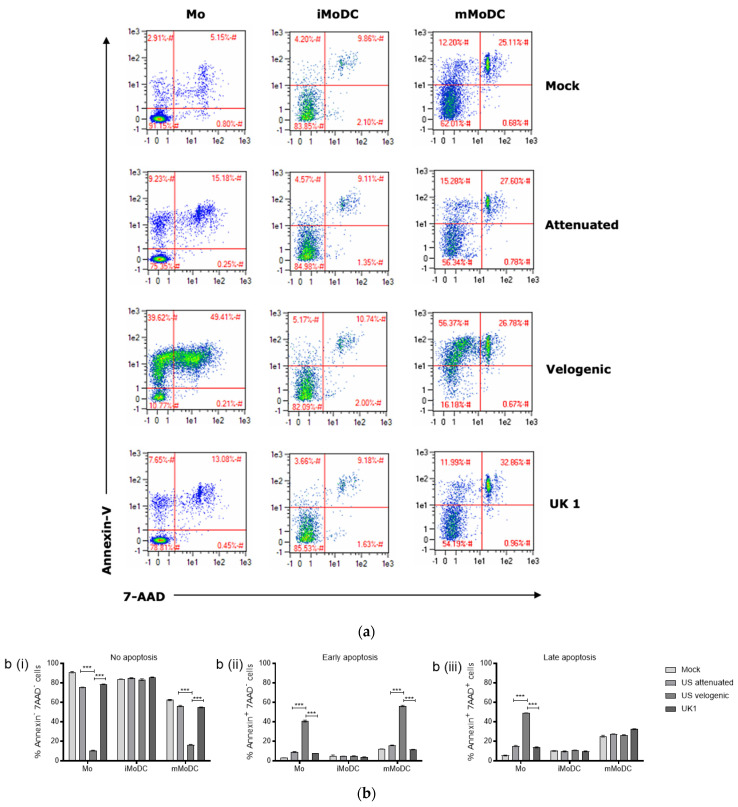
Apoptotic effect of EAV on Mo and MoDC. (**a**) Representative density plot of the apoptotic effect. The values in red represent the cell percentages in each gate. These plots are representative of nine independent repeats. (**b**) % Mo, immature (iMoDC) and mature MoDC (mMoDC) displaying no, early or late apoptosis. Mo, iMoDC and mMoDC were inoculated at MOI of 5 with different strains of live EAV. A mock control without virus was included. After 16 hpi cells were harvested and apoptosis assays performed by flow cytometry. Cells were stained with the apoptotic marker Annexin-V-FITC and live-dead stain 7-AAD-PE-Cy5 for 15 min at room temperature. Stained cells of the different treatments were analyzed by comparing the same number of events (10,000) in the monocyte or DC gate. A large percentage of Mo and mMoDC infected with the Velogenic strain were in the early apoptosis phase, Annexin-V^+^/7-AAD^−^ (**a**,**bii**). Mo infected with the velogenic strain displayed the highest proportion of cells in late apoptosis phase. Annexin-V^+^/7-AAD^+^ (**a**,**biii**). The viral apoptotic effect on iMoDC was minimal with most cells maintaining viability (Annexin-V^−^/7-AAD^−^) compared to Mo and mMoDC (**a**,**b**). Data in (**b**) are represented as the mean percentage apoptotic cells ± SEM (*n* = 4). Statistical significance was determined using a 2-way ANOVA with Bonferroni posttest to compare replicate means. *** indicate significant differences between sample means where *p* < 0.0001.

As it was established that EAV has the ability to infect and replicate lytically in Mo and MoDC, apoptosis was examined as a potential mechanism of cell death induced by EAV. The apoptotic process was assessed in infected Mo, iMoDC and mMoDC 16 h post infection (Figure 4a,b). The basal level of apoptosis varied within populations, and in the absence of infection cell populations contained a small percentage of apoptotic cells. Figure 4b show that the percentage expression of Annexin-V-FITC on Mo and mMoDC infected with the Velogenic strain significantly increased compared to mock, Attenuated and UK1 infected cells.

Monocytes and mMoDC infected with the velogenic strain displayed reduced viability (Annexin-V^−^/7-AAD^−^) with an early apoptotic phase (Annexin-V^+^/7-AAD^−^). However, only Mo infected with the velogenic strain were driven to late stage apoptosis and death (Annexin-V^+^/7-AAD^+^) compared to mMoDC, where there were fewer late stage apoptotic cells induced by the virus at 16 h p.i. There was no evidence that EAV causes necrosis (Annexin- V^−^/7-AAD^+^) in any of the cells. All virus strains, including the velogenic strain, were unable to induce apoptosis of iMoDC (Figure 4(bii,biii)). Compared to the velogenic strain, the attenuated and UK1 strains induced little apoptosis in Mo and mMoDC at this time point during primary replication. 

### 3.3. Phenotypic Changes in Mo and MoDC Induced by EAV Infection

The phenotype of Mo and MoDC correlates with some functions of these. Therefore, we analyzed the expression of key cell surface molecules under the influence of EAV by flow cytometry and qPCR. For Mo, key markers investigated were CD14, CD163 and CD172a. The DC molecules of interest for both the immature and mature states were CD206, CD83, MHC II and costimulatory molecules such as CD86, PD-L1, PD-L2, ICOS-L and B7-H3. All investigations were again carried out at 16 h post infection.

#### 3.3.1. Effect of EAV Infection on Live Mo and MoDC

**Figure 5:**
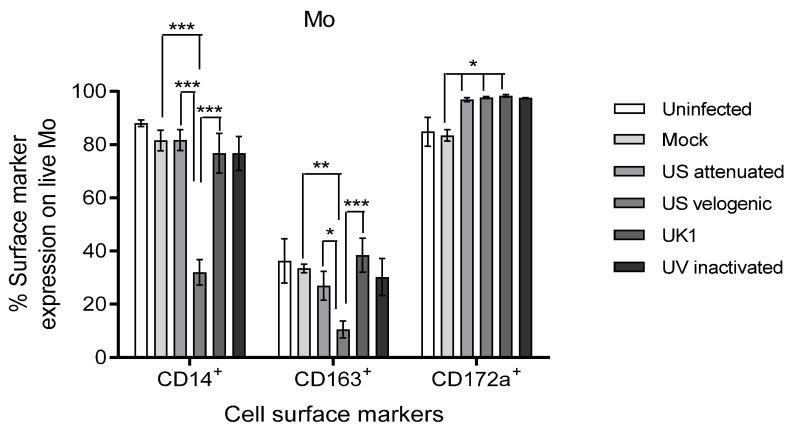
Phenotypic changes induced by EAV on live Mo. Monocytes were inoculated at MOI of 5 with different strains of live or UV inactivated EAV. Controls were uninfected (media only) and mock infected cells. Cultures were incubated at 37 °C in 5% CO_2_ for 16 h. Cells were stained with the live/dead fixable near-IR dead cell kit, followed by staining with anti-CD14-FITC, anti-CD163-Vioblue and anti-CD172-PE. Stained cells of the different treatments were assessed by comparing the same number of events (10,000) in the live cell target gate. The Velogenic strain significantly decreased the expression of CD14 and CD163 on Mo. There were no significant differences in the expression of CD172a; however, it was upregulated by all virus strains and the UV inactivated virus. Results were represented as the % surface marker expression ± SEM (*n* = 9). A 2-way ANOVA with Bonferroni post-test was used to compare replicate means. *, ** and *** indicate significant differences between sample means where *p* < 0.05, *p* < 0.01 and *p* < 0.001, respectively.

The velogenic strain significantly down-regulated the expression of CD14 and CD163 on live Mo compared to the attenuated and UK1 strains (Figure 5). Intriguingly, all EAV strains including the UV inactivated variant up-regulated the expression of CD172a. However, CD172a is constitutively expressed by most Mo already (around 75%). Together with the fact that there were no differences in the mean fluorescence intensity (MFI) of CD172a between the conditions, these changes were not considered significant. 

**Figure 6:**
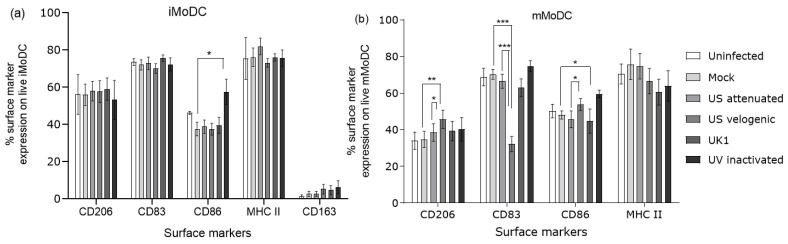
Phenotype of EAV infected live MoDC. Immature MoDC (iMoDC) and mature MoDC (mMoDC) were inoculated at MOI of 5 with different strains of live or UV inactivated EAV. Controls were uninfected (media only) and mock infected cells. Cultures were incubated at 37 °C in 5% CO_2_ for 16 h after which cells were harvested and stained to assess phenotype. Cells were stained with the live/dead fixable vioblue dead cell kit, followed by staining with anti-CD206-PE, anti-CD83-PECy5, anti-CD86-PECy5 and anti-MHC II-APC mAbs. Stained cells of the different treatments were assessed by comparing the same number of events (10,000) in the live cell target gate. (**a**) There were no biologically significant changes in the phenotype of EAV infected iMoDC. (**b**) The Velogenic strain significantly increased the expression of CD206 but decreased the expression of CD83 on mMoDC, thus rendering a more immature phenotype. There were no significant changes in the expression of CD86 and MHC II. Results were represented as the % surface marker expression of nine independent repeats ± SEM. A 2-way ANOVA with Bonferroni post-test was used to compare replicate means. *, ** and *** indicate significant differences between sample means where *p* < 0.05, *p* < 0.01 and *p* < 0.001, respectively.

EAV induced significant changes to the expression of surface molecules on mMoDC (Figure 6). The Velogenic strain induced upregulation of CD206 and a significant downregulation of CD83, while there were no significant changes in the CD86 and MHC II expression. In contrast, the different EAV strains did not induce significant changes in the surface molecules CD206, CD83, CD86 and MHC II on iMoDC (Figure 6a), which was in line with the low replication of EAV in these cells. Noticeably, the UV inactivated variant upregulated the expression of CD86 but not significantly against all controls. 

#### 3.3.2. Expression of Costimulatory Molecules EAV Infected Mo and MoDC

Changes in the expression of markers like CD206 and CD83 led to the investigation of further accessory molecules which influence antigen presentation, but for which there were no mAbs available to detect them. 

**Figure 7:**
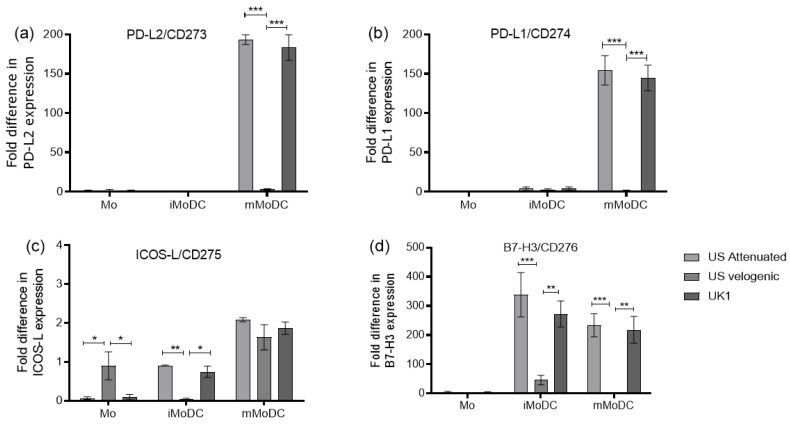
The expression level of costimulatory molecules on EAV infected Mo and MoDC. The co-stimulatory molecules, PD-L1/CD274, PD-L2/CD273, ICOS-L/CD275 and B7-H3/CD276, were assessed from EAV infected Mo and MoDC 16 hpi using duplex TaqMan qPCR assays with 18S rRNA gene as the reference. The normalized target gene expression was calculated by using the 2-ΔΔCt formula using the mock infected cells as comparator. There were significant differences in the expression of PD-L1/CD274 and PD-L2/CD273 on infected mMoDC with only the Attenuated and UK1 strains resulting in an upregulation (**a**,**b**). The overall expression differences of ICOS-L/CD275 on infected Mo and MoDC were low (less than 2-fold differences) (**c**). The expression level of B7-H3/CD276 on infected iMoDC and mMoDC was significantly different between strains (**d**). Results were represented as the average fold difference ± SEM (*n* = 3). A 2-way ANOVA with Bonferroni post-test was used to compare replicate means. *, ** and *** indicate significant differences between sample means where *p* < 0.05, 0.01 and 0.001, respectively.

**Figure 8:**
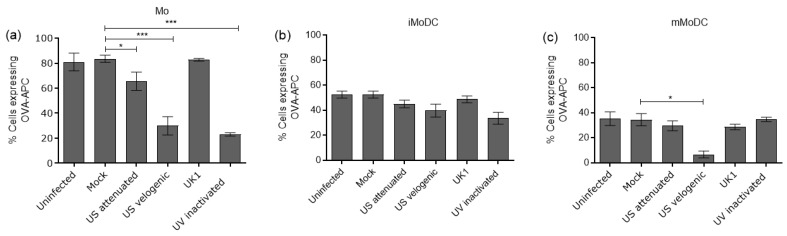
The capacity of EAV infected equine Mo and MoDC to endocytose antigen. Mo, immature MoDC (iMoDC) and mature MoDC (mMoDC) were inoculated at MOI of 5 with different strains of live or UV inactivated EAV. The percentage cells expressing OVA-APC* represented the difference in values obtained between the 37 °C and 4 °C (control). There were significant differences in the endocytic capacity of Mo and mMoDC infected with the Velogenic strain (**a**,**c**). There were no significant differences in the endocytic capacity of iMoDC infected with different EAV strains (**b**). The UV inactivated virus significantly reduced the uptake of OVA-APC* by Mo. Results are represented as the percentage positive cells ± SEM (*n* = 6). A 1-way ANOVA with Dunn’s post-test was used to compare replicate means. * and *** indicate significant differences between sample means where *p* < 0.05 and 0.0001, respectively.

All four molecules (PD-L1/CD274, PD-L2/CD273, ICOS-L/CD275 and B7-H3/CD276) investigated here are generally not expressed in monocytes. Only the velogenic strain led to a minimal increase in ICOSL/CD275 expression (Figure 7). Although ICOSL/CD275 was detected at low levels, it was the only marker where changes were observed in all three stages of myeloid cell differentiation; the molecule being upregulated by all virus strains on mMoDC. Intriguingly, all other costimulatory molecules were significantly more up-regulated by the attenuated and the UK field strain compared to the velogenic strain. In fact, only B7H3/CD276 was significantly up-regulated on iMoDC infected with the velogenic strain (Figure 8). For the three markers PD-L1/CD274, PD-L2/CD273, and B7-H3/CD276 expression was up-regulated more than 100× on mMoDC, but only B7-H3/CD276 was also upregulated (and to a similar degree) on iMoDC (Figure 7).

### 3.4. Changes Induced by EAV Infection on the Function of Mo and MoDC

Due to the roles Mo and DC play in the immune system, it was important to determine the impact EAV had on their function. Investigating their endocytic and phagocytic capacity assessed the ability of infected Mo and MoDC to take up antigen, whereas the stimulatory capacity of MoDC was investigated using the MLR assay.

#### 3.4.1. The Impact of EAV on the Endocytic and Phagocytic Capacity of Mo and MoDC 

Fluorescently labelled OVA was used to assess the endocytic capacity of infected Mo and MoDC. The data showed that the velogenic strain significantly reduced the ability of Mo and mMoDC to endocytose OVA (Figure 8a,c). Importantly though, there was no significant change in the endocytic capacity of infected iMoDC (Figure 8b). The attenuated and UK1 strains displayed similar results and, in most cases, did not differ significantly to mock. It should be noted that the UV inactivated variant decreased the ability of Mo to endocytose OVA.

**Figure 9:**
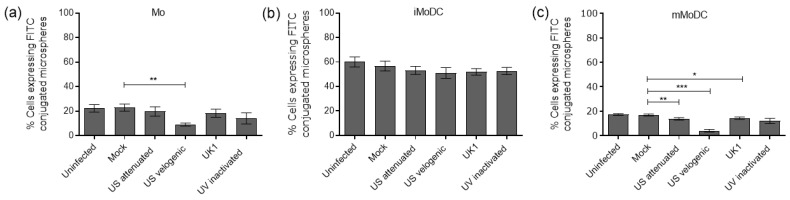
The capacity of EAV infected equine Mo and MoDC to phagocytose antigen. Mo, immature MoDC (iMoDC) and mature MoDC (mMoDC) were inoculated at MOI of 5 with different strains of live or UV inactivated EAV. The percentage cells expressing FITC-microspheres represented the difference in values obtained between the 37 °C and 4 °C (control). There were significant differences in the phagocytic capacity of Mo and mMoDC infected with the Velogenic strains (**a**,**c**). There were no significant differences in the phagocytic capacity of iMoDC infected with different EAV strains (**b**). Results are represented as the percentage positive cells ± SEM (*n* = 6). A 1-way ANOVA with Dunn’s post-test was used to compare replicate means. *, ** and *** indicate significant differences between sample means where *p* < 0.05, 0.01 and 0.001, respectively.

FITC-labelled microspheres were used to assess the phagocytic capacity of infected myeloid cells. The data reflect the results of the endocytic assays, i.e., the velogenic strain significantly decreased the phagocytic capacity of Mo and mMoDC, while the capacity of iMoDC was not affected (Figure 9). Here, the UV inactivated virus had little interference on phagocytosis (Figure 9b).

#### 3.4.2. The Ability of Infected MoDC to Stimulate T Cells

**Figure 10:**
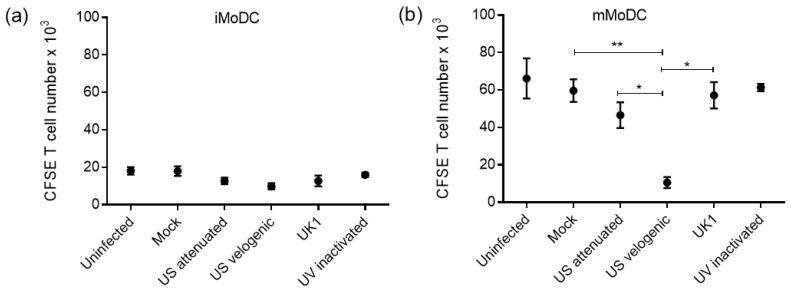
The ability of EAV infected equine MoDC to stimulate T cells in in vitro mixed leukocyte reactions (MLR). Immature MoDC (iMoDC) and mature MoDC (mMoDC) were inoculated at MOI of 5 with different strains of live or UV inactivated EAV. Cultures were incubated at 37 °C in 5% CO_2_ for 2 h prior to the addition of CFSE (FITC) labelled allogeneic T cells and further incubated at 37 °C for 3 days. Cells of the different treatments were analyzed using flow cytometry by comparing the same number of events (10,000) in the target gate. (**a**) There were no significant differences in the allostimulatory capacity of iMoDC infected with different EAV strains, although there was a trend in reduction. (**b**) There was a significant difference in the allostimulatory capacity of mMoDC infected with the Velogenic strain. Results are represented as proliferating T cell numbers ± SEM (*n* = 4). A 1-way ANOVA with Dunn’s post-test was used to compare replicate means. * and ** indicate significant differences between sample means where *p* < 0.05 and *p* < 0.001, respectively.

The allostimulatory capacity of infected iMoDC and mMoDC was investigated by in vitro MLR assays. Immature MoDC are known to be inferior to mMoDC in their ability to stimulate T cell proliferation as seen by the scale (Figure 10) and described [14]. The differences observed in allostimulatory capacity of infected iMoDC were not significant (Figure 10). However, the velogenic strain significantly reduced the allostimulatory capacity of mMoDC.

### 3.5. The Impact of EAV Infection on Differentiation and Activation

Once it was established that EAV inhibited some key functions of MoDC, it was important to analyze the effect of the virus on the differentiation and activation of MoDC. This was conducted by evaluating the capacity of infected monocytes to become immature MoDC and mature MoDC, respectively. The expression of the main equine DC markers namely CD206, CD86, CD83 and MHC II to determine differentiation and activation status [13,14] was measured by flow cytometry.

#### 3.5.1. Differentiation of EAV Infected Mo to iMoDC

**Figure 11:**
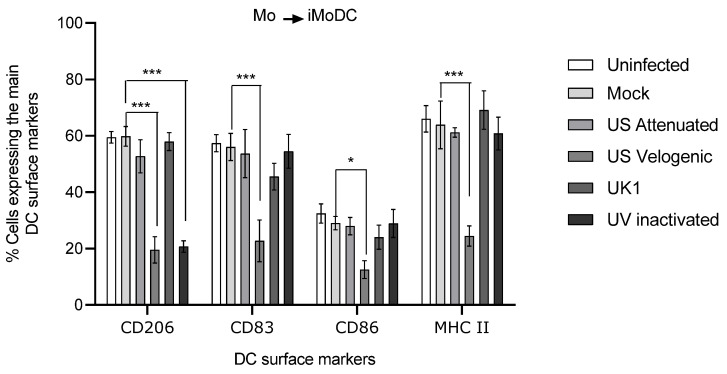
Effect of EAV infection on MoDC differentiation. Monocytes were inoculated at MOI of 5 with different strains of live and UV inactivated virus. Cells were incubated for 2 h after infection prior to the addition of 1000 U/mL of GM-CSF and 500 U/mL of IL-4. Cultures were incubated further for 16 h, after which cells were harvested and stained to assess phenotype by flow cytometry. For the different treatments, the same number of events (10,000) were analyzed in the target gate. There were significant differences during differentiation in the phenotype of MoDC infected with the Velogenic strain. Results were represented as the percentage surface marker expression ± SEM (*n* = 6). A 2-way ANOVA with Bonferroni post-test was used to compare replicate means. * and *** indicate significant differences between sample means where *p* < 0.05 and 0.001.

To assess the effect on differentiation, Mo were infected with EVA prior to the addition of cytokines. It was clearly demonstrated that the velogenic strain significantly reduced the expression of key surface molecules CD206, CD86, CD83 and MHC II (Figure 11), thus, the virulent strain inhibited the ability of Mo to differentiate into iMoDC. It is to be noted that the UV inactivated virus significantly reduced the up-regulation of CD206 on iMoDC. 

#### 3.5.2. Activation of EAV Infected iMoDC 

**Figure 12:**
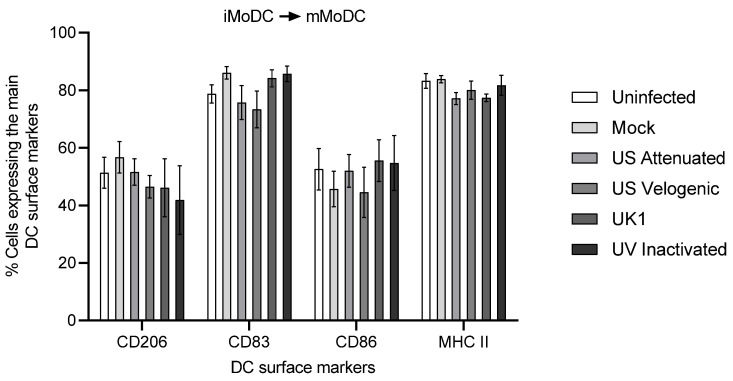
Effect of EAV infection on MoDC activation. Immature MoDC were inoculated at MOI of 5 with different strains of live and UV inactivated EAV. Cells were incubated for 2 h prior to the addition of the maturation cocktail. Cultures were incubated further for 16 h, after which cells were harvested and stained to assess phenotype by flow cytometry. For the different treatments, the same number of events (10,000) were analyzed by flow cytometry. Upon activation, there were no significant differences in the phenotype of MoDC infected with different EAV strains. Results were represented as the percentage surface marker expression ± SEM (*n* = 6). A 2-way ANOVA with Bonferroni post-test was used to compare replicate means.

The effect on activation was assessed by infecting iMoDC with virus prior to the addition of the maturation cocktail. Here, however, no significant changes in the phenotype of infected MoDC were observed (Figure 12). Accordingly, the virus had no effect on the ability of iMoDC to be activated.

### 3.6. Modulation of Cytokine Expression in Infected Mo and MoDC

Cytokines are important mediators of immunity. The effects of cytokines are paracrine and not restricted to infected cells. For this study, two cytokines were selected that are directly relevant to the anti-viral function of DC. IL-10 is known to block the transformation of iDC to immunogenic mDC and has been described extensively as a key factor in PRRSV, a related Arterivirus of pigs. IL-29 (IFNλ-1) in contrast is a positive regulator of the immune response. 

**Figure 13:**
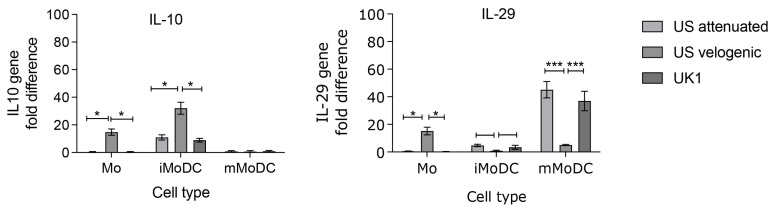
Cytokine expression levels in infected Mo and MoDC. The cytokines IL-10 and IL-29 were assessed in EAV infected Mo, immature MoDC (iMoDC) and mature MoDC (MoDC)16 hpi using duplex TaqMan qPCR assays with 18S rRNA gene as the reference. (**a**) IL-10 was significantly upregulated in Mo and iMoDC infected with the Velogenic strain. The expression in mMoDC was slightly above baseline levels for all strains. (**b**) The expression of IL-29 in Mo infected with the Velogenic strain was significantly upregulated, while in mMoDC its expression was increased but by only a 5-fold difference. There was no significant upregulation in iMoDC induced by the Velogenic strain. Results were represented as the average fold difference ± SEM (*n* = 3). A 2-way ANOVA with Bonferroni post-test was used to compare replicate means. * and *** indicate significant differences between sample means where *p* < 0.05 and 0.001, respectively.

The qPCR analysis revealed that IL-10 was significantly upregulated in Mo and iMoDC infected with the US velogenic strain (Figure 13a). However, the expression of IL-29 in infected Mo mirrored that of IL-10, in that the US velogenic strain significantly upregulated its expression (Figure 13b). Importantly though, there was no significant upregulation of IL-29 in iMoDC, whereas the expression was significantly upregulated in mMoDC infected with the attenuated and UK1 strains, which thereby exhibit a strong anti-viral bias upon infection. 

## 4. Discussion

Monocyte-derived dendritic cells (MoDC) represent a model of DC that contain all DC functions [12]. They are extensively used for clinical applications in medicine [31,32]. More so, they are also widely accepted to represent the inflammatory DC observed in vivo. 

### 4.1. Replication of EAV in Mo and MoDC

All viruses used in this study were concentrated and dialyzed to remove impurities that could have influenced the assays. Unsurprisingly, all three strains (the highly virulent US Bucyrus strain, an avirulent modification thereof and the low pathogenic UK1 field strain) have the ability to infect and replicate in Mo, iMoDC and mMoDC. However, the degree of replication varied significantly between cell types. Both Mo and mMoDC were highly susceptible, whereas iMoDC were fairly resistant to infection. In all cell types, the highly virulent Bucyrus strain showed the highest degree of replication corresponding to the pathogencity of the viral strains in vivo [16,19,21]. For monocytes, our data complement a previous study, which demonstrated the increased ability of the virulent Bucyrus strain and the reduced ability of a less pathogenic strain to infect CD14+ Mo [33]. Interestingly, Mo infected with the attenuated strain displayed a DC-like morphology 48 h post infection. It remains to be tested, however, if these cells contain any phenotypic and functional pattern of DCs without a cytokine driven differentiation. 

The ability of EAV to infect monocytes was attributed to the interactions among its envelope proteins, GP2, GP4, GP5 and M, which affected binding of the virus to its receptors. While the related arterivirus, PRRSV-1, has a very restricted tropism for porcine myeloid cells that express CD163 [34,35], EAV has a broad cell tropism which has been attributed to the interaction of the minor envelope proteins which play a crucial role in viral attachment [36]. Since less than half of equine Mo expressed CD163 and MoDC rather none at all (Figure 5 and Figure 6), CD163 may be implicated in the adhesion of EAV to target some cells, but a receptor for EAV identified subsequent to this study (CXCL16, [37]) needs further investigation. Given the broad host range of EAV and the restricted expression of CXCL16 on haematopoetic cells such as some DC or a subset of T cells, CXCL16, however, is almost certainly not the only receptor used by EAV. 

It is possible that iMoDC do not possess the necessary EAV receptor(s) for EAV infection, but this hypothesis seems unlikely as both monocytes and mMoDC were highly susceptible to infection. Moreover, the highly pathogenic Bucyrus strain did show a significant (50-fold) increase in the ORF1 gene expression, but the titre in the supernatant remained low compared to Mo and mMoDC. It is thus plausible that the immature DC state has the ability to limit the virus replication. Further studies can use the cell systems established here to determine the EAV receptors on both Mo and mMoDC, and to determine restriction factors occurring in iMoDC. 

Apoptosis is a cell death mechanism through which no inflammation is triggered, thus viruses can better hide from the immune system if they induce apoptosis as part of their lytic replication. Conversely, the induction of apoptosis is a mechanism by which cells can limit replication of viruses. A study investigating the replication of PRRSV in alveolar MΦ has shown the death of these by apoptosis [38]. EAV infected Mo and mMoDC clearly underwent apoptotic death induced at the end of a replication cycle. Further time kinetic assays must be conducted to assess if the induction of anti-apoptotic strategies sustain cell survival during replication, as shown for many viruses, including PRRSV [38]. 

The present study only allows for a limited set of conclusions regarding the impact of apoptosis induced by EAV strains. What seems clear is a correlation of pathogenicity with the induction of apoptosis as the impact of velogenic strain clearly stands out from both the UK1 and the attenuated Bucyrus one (Figure 4). The significant cell death induced in monocytes in particular means that monocyte-derived cell differentiation (both MoMF and MoDC) would be impacted. 

More speculative, but likely, would be the correlation of early apoptosis in mMoDC infected with particularly the velogenic strain (Figure 4) and the reduced functional activity of mMoDC in both endocytosis/phagocytosis (Figure 8 and Figure 9) and T cell stimulation (Figure 10). In vivo, however, it is particularly immature DC that are supposed to endocytose antigen, process and present while undergoing maturation to mature DC. This process would not seem to be particularly affected by apoptosis using MoDC as a model (Figure 4 and Figure 12). 

### 4.2. Modulation of DCs by EAV Infection

The phenotype displayed by infected Mo and MoDC revealed no significant changes in the surface marker expression on iMoDC, whereas there were significant changes in cell surface markers on Mo and mMoDC induced by EAV, reflecting an altered state of cells post infection. 

DC play a central role in priming T cell responses [8,39] to generate effective cell-mediated immunity against viruses [40]. Activation of T cells starts with the acquisition of antigen and then requires two signals: one is antigen specific through the interaction of MHC II and T cell receptors, and the second is a costimulatory signal which is non-specific but can be both activating and de-activating. To assess the impact of EAV infection on the function of DC several assays were performed. 

Endocytosis in human DC is associated with several receptors, among them the mannose receptor CD206, the scavenger receptor CD163, and the LPS co-receptor CD14 [41,42,43,44,45]. CD163 was expressed on some Mo and downregulated upon infection with the Velogenic strain as was the expression of CD14. 

The endocytic capacity of equine Mo infected with the Velogenic strain was reduced. However, since the UV inactivated virus also decreased the endocytic capacity of Mo and iMoDC, the reduction observed with the Velogenic strain may not only be due to replication. Further investigations are required to better understand the antigen uptake pathways in equine Mo and MoDC. 

The upregulation of CD206 on mMoDC infected with the Velogenic strain indicates a decreased maturation, while the expression of MHC II was not affected by the infection of EAV; hence, these cells were still able to present antigen and deliver the first signal of antigen presentation. However, the expression of CD83 has been associated with the ability of DC to stimulate T cell proliferation [46,47]. The downregulation of this maker on mMoDC infected with the Velogenic strain can be linked to a reduced ability of these cells to prime T cells. 

PD-L1 (CD274) and PD-L2 (CD273) are molecules which, restricted to mature DC, are able to inhibit T cells [48]. For horses it had been proposed that the downregulation of PD-L1 and PD-L2 on uninfected mMoDC is associated with an inhibitory role in the equine immune response [14]. However, the biological role of PDL1 and PDL2 is likely more complex. It was shown that PD-L2 trigger IL-12 production in murine DC thus activating T cells [49]. It has since been demonstrated that PDL1 in early immune reactions correlates rather with immune cell activation [50]. More so, the expression of PD1 on tissue-resident memory T cells (TRM) does not affect their functional abilities [51]. In this light the strong activation of both PDL1 and PDL2 by attenuated and less pathogenic EAV strains seems to rather support the activation of the immune system. 

ICOS-L (CD275) is another member of the B7 family with homology to CD80/86 that has been shown to activate T cells via ICOS—a molecule with homology to CD28. ICOS/ICOS-L interaction seems to shift the immune response to the Th2 type. Its sustained low expression in the presence of both the Attenuated and Velogenic strains implies that it is not targeted by EAV. 

B7-H3 (CD276) still remains enigmatic in its ambivalent functions. B7-H3 was initially considered necessary for T-cell costimulation [52]. B7-H3 is now assumed to have a predominantly inhibitory role, suppressing T-cell activation and B7-H3 on DC reduces CD4^+^ and CD8^+^ T-cell activation, as well as effector cytokine release by inhibiting the expression of major transcriptional factors, such as NF-κB, while expression in tumour cells has been shown to suppress NK cell activation [53,54]. The expression of B7-H3 was upregulated roughly 300-fold in the presence of the Attenuated strain and UK1 compared to the Velogenic strain in both iMoDC and mMoDC. Hence, strains that do not kill the cell seem to employ other ways of downmodulating the immune response. 

In most cases it was observed that the UV treatment of the virus did not affect phenotype and function of Mo and MoDC. However, it did induce few phenotypic changes, including the upregulation of the costimulatory molecule CD86 on MoDC. This molecule plays a key role in T cell activation and MoDC in the presence of inactivated virus were still able to induce activation of T cells. The significance of this effect is unknown, but probably indicates that the inactivated virus is still capable of activating TLRs.

Immature MoDC were phenotypically unaffected and still functionally active in the presence of different EAV strains. Mature MoDC infected with the low pathogenic strains too were largely unaffected and will contribute to the development of an immune response. It is well documented that CD8^+^ cytotoxic T lymphocytes (CTL) play a key role in the clearance of viral infections and a previous study specifically implicated these to target EAV-infected cells [55]. Indeed, iMoDC and mMoDC infected with less pathogenic strains may still have the ability to stimulate CD8^+^ T cells leading to clearance of these infected cells. 

It must also be noted that a previous study has demonstrated the ability of EAV to infect CD3^+^ T cells [33]. Here it is possible that equine DC may have the ability to transmit the virus to T cells. The ability of both the immature and mature DC states to transmit the virus to T cells will need to be investigated. 

IL-10 and IL-29 (IFNIII) belong to the IL-10 superfamily and have been previously shown to be expressed by monocytes, macrophages and dendritic cells [56,57]. Hence, we considered their expression under infection relevant since both cytokines might be able to skew an immune reaction. 

IL-10 has multiple functions, but importantly is a known immune-modulator that is secreted by iTreg, TLR-activated macrophages and can inhibit DC differentiation and activation. Accordingly, it can limit T cell responses, such as described in Lymphocytic Choriomeningitis virus (LCMV) infection [58]. IL-29 sits at the border of the IL-10 superfamily and is also known as Interferon lambda 1 (IFNλ1) [48,57,59]. Accordingly, IL-29 supports the anti-viral response against viruses [60,61]. 

Indeed, Velogenic EAV specifically upregulated IL-10 in Mo and mMoDC compared to other EAV strains, and this effect was not observed in iMoDC which it infects least. This is of importance since IL-10 in a paracrine way is then able to stop the maturation of DC, thereby limiting the immune response, including against secondary infections. Conversely IL-29 was mainly detected in mMoDC after treatment with the attenuated EAV preparation or the less virulent variant of UK1. Accordingly, the latter are well able to induce an anti-viral response, whereby such is not the case for infection with Velogenic EAV, which supports the phenotype of both viruses.

### 4.3. EAV in Monocytic Cells Suppressed Their Differentiation into DC

While some viruses such as HCMV inhibit the differentiation of DC [62], other viruses such as VSV, VV and influenza A viruses seem to induce the rapid differentiation of Mo [63]. In line with the above results, the Velogenic strain inhibited the ability of Mo to differentiate towards iMoDC. Equine Mo treated with the attenuated and UK1 strains still had the ability to differentiate into iMoDC. However, further studies to determine if these cells are fully functional will be required in the future. 

As for the differentiation, some viruses such as HCMV and Japanese encephalitis virus (JEV) suppressed maturation of human and mouse DC [64,65]. In contrast, Dengue virus stimulation of human DC rather drives the maturation of cells [66], which supports the pathogenesis of Dengue fever. Here, infected equine iMoDC seem to take a neutral position, i.e., the limited replication of iMoDC did not hinder their activation, which in turn provides in mMoDC a very good target cell for EAV. 

In summary, it has been clearly demonstrated that mMoDC are highly permissive to infection while iMoDC are less susceptible. Through changes upon infection of mMoDC, EAV disrupts the adaptive immune response from antigen uptake to stimulating T cells. Monocytes are more susceptible than iMoDC but less than mMoDC. In particular, the pathogenic strain reduced the endocytic and phagocytic capacity of Mo and mMoDC and inhibited the ability of mMoDC to stimulate alloreactive T cell responses. 

While MoDC resemble a useful model to study the interactions of DC and EAV in vitro, they only represent one type (inflammatory DC) of the various DC types occurring in vivo in blood and different tissues. Hence, future work will be required to complement this with identifying target myeloid cells ex vivo and in situ. 

## Figures and Tables

**Table 1:** Primer and probe sequences used.

Gene	Primer and Probe Sequence 5′ →3′	Source
EAV ORF1	TAGCCATTGAAGAGGCAAGT ^a^	in house
	GGCAAAAGTTTTAACCAGCA^b^	
	6FAM- GACCACGCGTCTGCTAAGCG- BBQ ^c^	
IL-29	GGCAGGTTCCAATCTCTGTC ^a^	in house
	CAGCGTCAGGTGTAGCTCAG ^b^	
	6FAM- TCTTCCCCATGACCAGAGAC- BHQ-1 ^c^	

^a^. forward primer sequence; ^b^. reverse primer sequence and ^c^. probe sequence. The EAV ORF-1 PCR is based on an alignment of available sequences in GenBank; the IL-29 PCR was designed using GenBank accession no XM_001501189.

## Data Availability

Not applicable.
